# Amoxicillin/Clavulanic Acid-Induced Agranulocytosis: A Case Report and Review of the Literature

**DOI:** 10.1155/2019/5718716

**Published:** 2019-10-23

**Authors:** Aristides Armas Villalba, Marya Haq, Cunxian Zhang, Jinen Thakkar

**Affiliations:** ^1^Internal Medicine Department, Kent County Hospital, Warren Alpert Medical School of Brown University, Providence, RI, USA; ^2^Associate Professor of Pathology and Laboratory Medicine, Brown University, Providence, RI, USA; ^3^Chief of Pathology, Kent County Hospital, Warwick, RI, USA

## Abstract

Amoxicillin/clavulanic acid is one of the widely prescribed antibiotics in the outpatient setting giving excellent antimicrobial coverage and relatively safe profile in terms of adverse reactions. Gastrointestinal and cutaneous reactions are among the commonly reported. Our goal is to describe a case of agranulocytosis induced by amoxicillin/clavulanic acid in a previously healthy patient and review prior reports with similar presentation.

## 1. Introduction

According to the Center for Disease Control and Prevention (CDC), 269.4 millions of antibiotic prescriptions were ordered in the outpatient setting within the United States of America in 2015. Amoxicillin is the most commonly prescribed antibiotic, and amoxicillin-clavulanate is the third most commonly prescribed [1].

There are drugs classically described as the potential cause of neutropenia or agranulocytosis, but amoxicillin/clavulanate is not included in the list. Since the development of the drug, the most common side effect or adverse reaction reported is diarrhea.

Here, we present a case about amoxicillin/clavulanate-induced agranulocytosis and review of the literature.

## 2. Case Report

A 49-year-old male patient without a significant past medical history presented to hospital with complaints of diarrhea. Five days prior to presentation, he developed a sore throat and consulted a local urgent care center where rapid influenza and streptococcal antigen testing were completed. Despite the negative results of both of these tests, he was prescribed amoxicillin/clavulanate (875/125 mg twice a day, day 5 on presentation to the hospital).

His symptoms persisted and progressed, with the addition of watery diarrhea to his complaints, at which point he presented to the emergency department. Initial physical exam was significant for a temperature of 38.7°C (101.7°F), with other vital signs within normal limits. Skin exam demonstrated a generalized macular and erythematous exanthema. The pharynx was found to be erythematous but without exudates, and chest auscultation demonstrated bilateral wheezing. The remainder of the physical exam was unremarkable.

Initial laboratory data reported hemoglobin 14.0 g/dL, hematocrit 40.6%, platelet count of 159 × 10^3^/mm^3^, and white blood cell count of 500/mm^3^. Considering the fever, physical examination and degree of neutropenia, the patient was admitted to the hospital with diagnosis of febrile neutropenia and started on broad-spectrum antibiotics immediately (vancomycin and piperacillin-tazobactam).

**Figure 1:**
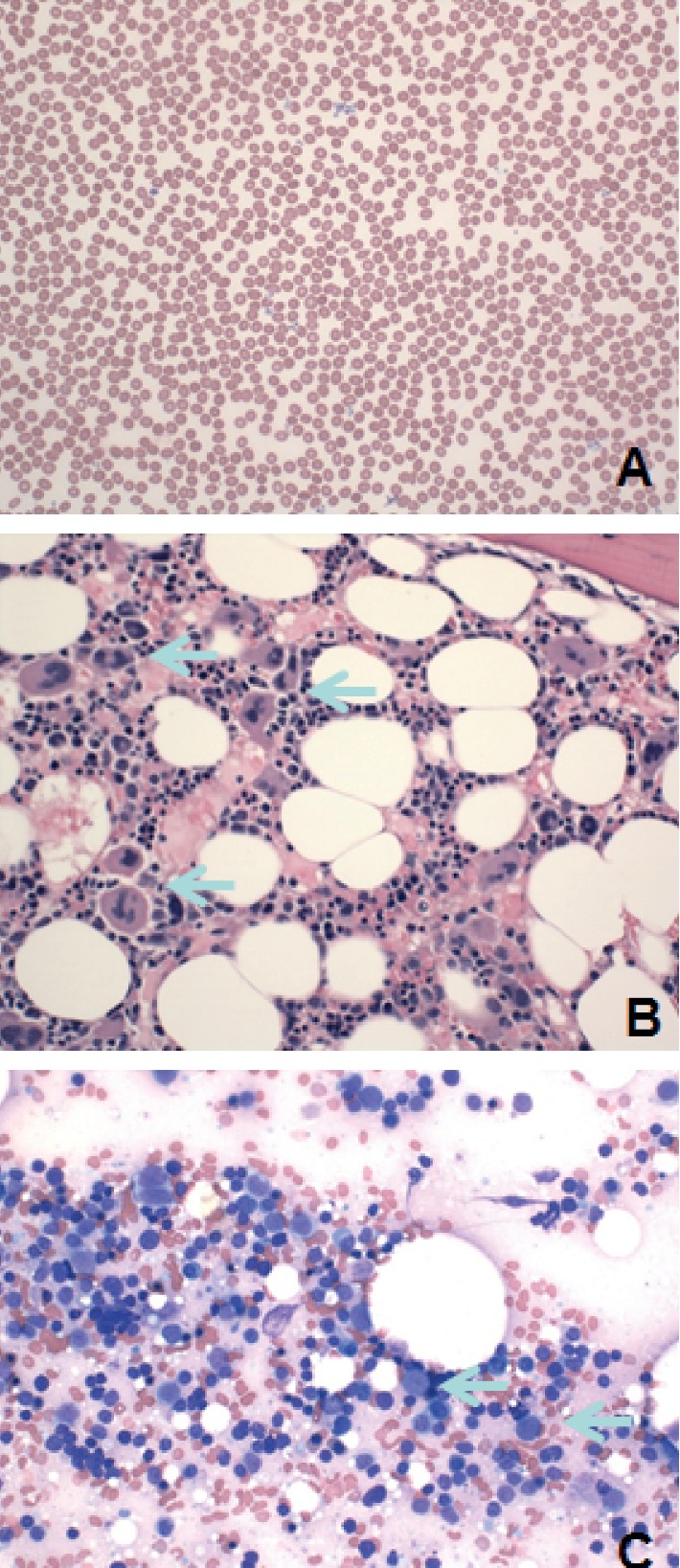
Peripheral blood smear and bone marrow studies. (a) Peripheral blood smear shows severe neutropenia with no neutrophils seen in this field (hematoxylin and eosin stain; ×400), (b) bone marrow biopsy with normal cellularity, increased megakaryocytes (blue arrows), and severe decrease in neutrophils (hematoxylin and eosin stain; ×400), and (c) bone marrow smear displays severely decreased segmented granulocytes and increased blasts (blue arrows; Wright–Giemsa stain ×400).

At the same time, blood cultures were drawn and the patient was started on filgrastim (granulocyte-colony stimulating factor) and continued on a daily basis. When he remained febrile and profoundly leukopenic, the hematology department was consulted for bone marrow aspirate and biopsy. On day three, marrow examination was done and reported a 40–60% cellularity with myeloid hypoplasia (myeloid to erythroid precursor ratio of 0.5 : 1) and increased blasts (Figure 1).

**Figure 2:**
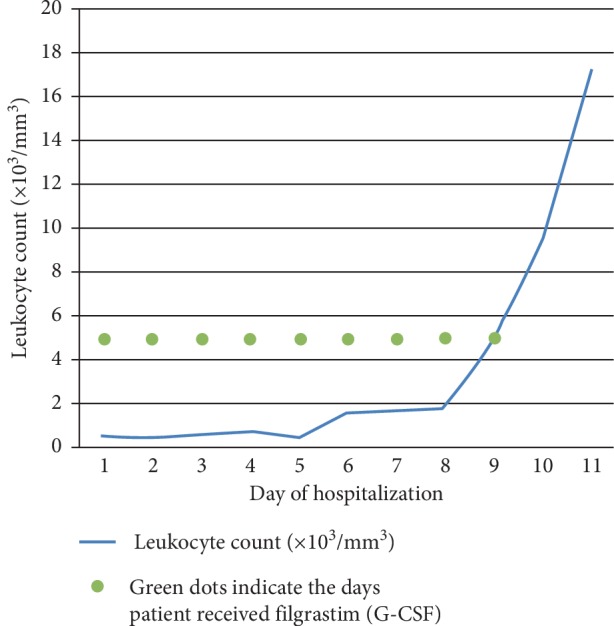
Daily leukocyte trend throughout the admission in the hospital.

Despite a slowly rising leukocyte count, he continued to spike fevers for the first seven days of admission; on day nine of admission, his leukocyte count had normalized (Figure 2). Throughout the stay in hospital, extensive work-up for infectious and immunologic causes of agranulocytosis (Table 1) was done and revealed only a positive IgG result against parvovirus B19. Since the diagnosis was still a challenge, skin biopsy of the aforementioned rash was performed and reported perivascular and periadnexal lymphoplasmacytic inflammation.

His clinical condition continued to improve, with stable vital signs including lack of recurrence of fevers for the last four days of his inpatient stay, and as there were no further indications for either antibiotics or filgrastim, he was discharged home on day eleven of his hospital stay.

One week after discharge, he was seen in follow-up by his hematologist, with continued demonstration of a complete blood count within normal limits and no further concerning signs or symptoms.

## 3. Discussion

Classically, neutropenia is defined as an absolute blood neutrophil count less than two standard deviations below the normal mean, and agranulocytosis is often used to define severe neutropenia (below 0.5 × 10^9^ cells/liter) [2]. The causes of neutropenia can be classified as a disorder in the production and distribution or turnover of neutrophils.

In terms of duration, neutropenia can be classified as transient or chronic, with three months being the cutoff point. The most common etiology of transient neutropenia is a viral infection [3].

Several studies have studied the incidence of drug-induced neutropenia, estimating it anywhere between 1.1 and 15.4 cases per million population around the world per year [4–6].

Interestingly, despite an extensive infectious work-up, no infectious etiology was identified in this patient with acute onset agranulocytosis or neutropenia. He did present to the emergency department six days after the onset of symptoms, but most infectious causes of agranulocytosis do not typically cause the persistent marrow suppression seen in this patient despite resolution of his symptoms.

Taking the extensive work-up, the presence of a maculopapular rash and the temporal association with exposure to amoxicillin-clavulanate, we concluded that his presentation was consistent with amoxicillin-clavulanate-induced agranulocytosis. On top of that, ferritin and C-reactice protein levels were found to be elevated, well know acute phase reactants that can be triggered by immune processes.

There are well-described nonchemotherapeutic medications associated with agranulocytosis, and the term is known as idiosyncratic drug-induced agranulocytosis.

There are only two case reports associating amoxicillin-clavulanate with agranulocytosis, but unfortunately the investigations were not as thorough as our patient had, leading to multiple questions about whether it truly was the drug that caused the hematologic findings [5].

Briefly, the pathogenesis of the disease has been attributed to direct “toxic” effect of the medication (or a metabolite) and recently has been described an immune-mediated process. A widely accepted hypothesis explains that the drug can activate the immune system via haptens that later will induce direct damage to the cell or bone marrow itself [7, 8].

Our approach to this patient's management was to first exclude infections as a possible etiology, and the extensive work-up can be seen in Table 1. In the meantime, broad-spectrum antibiotics and granulocyte-colony stimulating factor were administered to the patient as part of the treatment for neutropenic fever.

The patient continued to be severely neutropenic despite multiple treatments with granulocyte-colony stimulating factor (G-CSF). There are several hypotheses proposed to explain the phenomenon: the first one is an immune-mediated reaction after the patient is exposed to the drug and the second one, the drug itself inducing a toxic microenvironment in the marrow that inhibits the production of new cells [9].

Four days after G-CSF was started, the bone marrow was still not responding and bone marrow biopsy and aspirate was performed. The pathology department reported 40–60% cellularity with marked myeloid hypoplasia. At the same time, skin biopsy was taken as well for further understanding of the rash and reported perivascular and periadnexal lymphoplasmacytic inflammation. Both of these findings are consistent the with drug-related reaction.

We conclude that over the years, amoxicillin-clavulanate has demonstrated to be a safe drug that has been widely used. Despite the two prior reports and ours, prescription must be continued if there is a formal indication in the appropriate patient to avoid harming the patient and reducing antibiotic resistance rates as much as possible.

## Figures and Tables

**Table 1:** Infectious and immunologic work-up performed during the admission.

Test	Result	Comments
Chest X-ray	Normal examination	
CT chest, abdomen, and pelvis	Unremarkable	Simple cysts in the left kidney.
Blood cultures	Negative	Performed on three different days (two sets each)
Stool analysis	C. albicans isolated in the culture	Negative for bacteria, ova, and parasites.
Influenza rapid diagnostic test	Negative	
Rapid strep test	Negative	
Mononucleosis spot test	Negative	IgM heterophile antibody
Urine analysis	Negative for infection	
Blood parasites	Negative	
Viral hepatitis	Negative	Hepatitis A, B, and C.
Cytomegalovirus	Negative	IgG and IgM
Human immunodeficiency virus	Negative	Elisa and PCR
Parvovirus B19	IgG positive	IgM negative
Babesiosis panel	Negative	PCR for B. microti, B. duncani, and B. divergens.
T. pallidum antibodies	Negative	
Respiratory virus culture	Negative	Reported after 14 days of incubation
Fungitell	Negative	
B. burgdorferi	Negative	IgG and IgM
Rheumatoid factor	Negative	
Anti-nuclear antibodies	Negative	
Ferritin	615 ng/mL	Normal values from 18 to 270 ng/mL
Procalcitonin	0.12 *μ*g/mL	Normal values from 0 to 0.26 *μ*g/mL
C3 level	138 IU	Normal values from 88 to 200 IU
C4 level	37.2 IU	Normal values from 16 to 47 IU
C-reactive protein	17.4 mg/dL	Upper limit of normal 0.5 mg/dL
